# Supplementary material for: Are carbon emissions trading and green financial instruments synergistic? -Comprehensive quantitative research based on content analysis
Source: PLoS One. 2024 Mar 7;19(3):e0298601. doi: 10.1371/journal.pone.0298601 (PMC10919601; doi:10.1371/journal.pone.0298601)
Supplement: S1 Data — (ZIP) [file pone.0298601.s001.zip › Data/地级市Z_F工作报告.zip!/╔╜╢1⁄2/╫═▓⌐╩╨/└┤╫╘╣1⁄2╓┌║┼í╛┬φ┐╦╩2╛▌═°í┐_2019.docx]

各位代表：

现在，我代表市人民政府，向大会作政府工作报告，请予审议，并请各位政协委员和列席会议的同志提出意见。

一、2018年政府工作回顾

刚刚过去的一年，我们坚持以习近平新时代中国特色社会主义思想为指导，深入贯彻党的十九大和习近平总书记视察山东重要讲话、重要指示批示精神，牢牢把握稳中求进工作总基调，在中共淄博市委的坚强领导下，紧紧围绕“一个目标定位、四个着力建设、十个率先突破”总体思路和工作布局，按照市十五届人大三次会议确定的目标任务，团结实干，攻坚克难，担当作为，全市经济社会发展稳中有进，城市综合实力持续提升。预计地区生产总值迈上5000亿元台阶，同比增长6.5%左右；一般公共预算收入完成385.23亿元，同比增长6.54%；城镇居民和农村居民人均可支配收入分别达到42270元、18270元，同比增长7.3%左右和7.8%左右。

（一）新旧动能转换提质加速。制定实施新旧动能转换重大工程规划和推进方案，聚力做好“高新轻绿”四篇文章，市重大项目完成年度投资1000亿元，新开工工业项目中新兴产业项目数量占到72%，现代服务业占服务业比重提高到50.3%。扎实开展“技术改造突破年”，实施重点技改项目363个，传统产业智能化信息化绿色化改造呈现整体发力态势，预计工业技改投资同比增长28%，规模以上工业主营业务收入、利润同比分别增长13%和20%。启动“两城一谷”规划建设，新增省级以上创新平台61家，高新技术企业预计新增100家以上，同比增长120%左右，研发投入占比和高新技术产业产值占比分别达到2.46%和39%左右，7项科研成果荣获省科学技术一等奖。经济开发区体制机制改革深入推进，6个化工园区获省政府认定，建陶产业创新园一期工程建成投用。优质企业群体不断壮大，精准培训企业管理者2.7万人，新增规范化改制企业300家、齐鲁股交中心挂牌企业74家，4家企业入围中国企业500强、7家企业入围中国民营企业500强，以中小企业为主体的省级特色产业集群达到12个。全面落实“人才新政23条”，新增院士、“千人计划”专家等高端人才50名，新落户本科生、研究生4936人，同比增长109%。

（二）三大攻坚战扎实推进。坚定不移抓好中央、省环保督察“回头看”以及生态环境部强化督查反馈问题整改，全面启动“四减四增”三年行动和生态环保十个专项行动，完成大气污染治理任务2383项，全市PM10、PM2.5、SO2、NO2浓度同比分别改善10.9%、12.7%、36.8%、8.5%。压减粗钢产能70万吨、生铁产能60万吨，削减煤炭消费72万吨，入选2018年北方地区冬季清洁取暖试点城市。深入开展绿色矿山建设和关闭矿山地质生态修复，全面完成“3322”生态绿化工程，孝妇河湿地公园、齐盛湖公园二期、文昌湖环湖公园建成开放。突出抓好范阳河治理等135项水污染防治重点工作，主要河流断面COD和氨氮浓度稳定达标，全省河长制湖长制现场推进会在我市召开，创建为国家节水型城市。严格规范政府性债务管理，完善财政预算管理机制，财政经济保持健康运行。实施金融风险防控三年攻坚，多措并举化解了33个重点担保圈、490户企业信用风险。脱贫攻坚实现重要进展，黄河滩区居民迁建工程加快推进，474个省扶贫工作重点村全部达标退出，市现行标准下贫困人口基本实现“两不愁三保障”。

（三）城市现代功能明显提升。全省“多规合一”试点及城市发展战略规划、总体规划编制进展顺利。全面启动火车站南广场改造建设，柳泉路改造、北京路北延等12个骨干道路工程如期竣工，6座城市立交有序推进；济青高速、滨莱高速、G309青兰线改扩建等重点交通工程加快实施，G205淄博收费站等4个普通国省道收费站实现撤站；济青高铁及淄博北站、临淄北站建成通车，张东铁路电气化改造全面完成。着力实施引黄供水配套、刘征水源地、第二环保热电厂、4座城镇污水处理厂以及总投资23亿元的骨干电网建设改造等公用重点工程，城市承载力不断提升。做实城市精细化管理体制，深化市容环境专项治理，荣获“山东省适宜人居环境奖”。丰富提升城市文化内涵，61个文旅融合重点项目稳步推进，成功举办齐文化节、世界足球文化高峰论坛系列活动，城市规划建设更多融入齐文化等地域文化元素。市文化中心全面建成，淄博中国陶瓷琉璃馆、淄博大剧院等一批高品位文化设施正式启用。新建7个区县档案馆，6家图书馆获评国家一级图书馆，我市蝉联文化强省建设先进市。巩固提升全国文明城市创建成果，城市现代化风貌进一步彰显。

（四）乡村振兴战略顺利实施。制定出台全市乡村振兴规划及五个专项工作方案，市级以上农业龙头企业达到178家，培育发展千禧农谷、天然富硒农业产业园等一批“新六产”项目，太河片区等10个乡村旅游连片发展区初具规模。粮食生产战胜自然灾害获得丰收，粮食安全保障能力明显提升。健全农村公共服务体系，实施乡村教师提升工程，“第一村医”覆盖920个村庄，镇村公共文化服务设施实现全覆盖。深入推进美丽乡村建设，完成135个村庄环境综合整治，改造农村危房1.15万户，新改建农村公路506公里，完成545个村“户户通”硬化任务，改造农村旱厕7.7万户，全国农村改厕工作推进现场会、全省乡村振兴暨脱贫攻坚（中片区）现场会在我市召开。

（五）深化改革开放成效显著。全面启动市县机构改革。围绕打造“三最”城市升级版，聚焦“一次办好”改革，组织实施优化企业开办、信息资源共享等12个专项行动，新登记市场主体7.6万户，“不见面审批”“一门办理、一窗受理、一网通办”新机制加快建立，全省深化“一次办好”改革现场会在我市召开。持续深化财税、投融资、公共资源交易、国有企业、事业单位、统计管理、农村集体产权制度等重点领域改革，进一步丰富了全面深化改革“淄博实践”品牌内涵。扎实推进“双招双引”和“四大招引工程”，成功举办首届齐商大会以及陶博会、新材料技术论坛等重要展会，精准组织一系列境内外招商活动，新增世界500强及行业领军企业项目11个，到位外来投资同比增长18.5%，蓝帆医疗等企业海外并购实现重大突破。与驻淄央企省企合作取得积极进展，中铝齐鲁工业园等55个企地融合项目顺利推进，完成年度投资42亿元。积极参与“一带一路”建设，成为“齐鲁号”欧亚班列4个首发城市之一，全市货物贸易进出口总额同比增长35.7%，其中出口增长11.8%，新兴服务贸易增长40%以上，博山区入选首批国家文化出口基地，我市居“中国外贸百强城市”第60位。

（六）民生社会建设持续加强。落实积极就业创业政策，城镇登记失业率2.44%。大力推进“全民参保计划”，参加各类保险人数达1031.7万人次。进一步提高社会救助水平，做好特困人员集中供养，全面落实“双拥”优抚安置政策。实施消除“大班额”规划（2018-2020），新改扩建幼儿园145所、中小学18所，国家义务教育优质均衡县（区）创建稳步推进。市中心医院西院区等医疗重点项目基本建成，老年人家庭医生签约率达到81%，基层卫生服务机构标准化率达到83%。扎实开展迎接国家卫生城市复审、全国基层中医药工作先进市创建工作。改造提升165个城乡社区服务中心，新建城市社区日间照料中心30家、农村幸福院106家。全面完成“三改三建”年度任务。创建为“山东省食品安全市”。参加十八届亚运会和二十四届省运会取得优异成绩。顺利完成消防救援队伍改革转制。国防动员和后备力量建设稳步推进。大力加强安全风险防控和隐患排查治理，未发生较大以上生产安全责任事故。实施社区治安综合治理精细精准工程，深入开展扫黑除恶专项斗争，圆满完成青岛上合峰会等重大活动安保维稳任务，社会大局保持和谐稳定。物价、广播电视、民族宗教、外事侨务、对台、防震减灾、人防、气象、史志、档案、仲裁、住房公积金、知识产权、科协、关心下一代、妇女儿童、青少年、老龄、慈善、残疾人、红十字、对口支援等各项事业都取得了新成绩。

一年来，我们高度重视政府自身建设，深入学习贯彻习近平新时代中国特色社会主义思想和党的十九大精神，牢固树立“四个意识”，坚定“四个自信”，坚决做到“两个维护”，在思想上政治上行动上始终同以习近平同志为核心的党中央保持高度一致。持续深化政府系统党的建设，坚决抓好中央和省委巡视反馈意见整改落实，认真组织开展“大学习、大调研、大改进”“新时代、新理念、新担当”等系列活动，深入推进“三个转变、一个强化”，着力解决“四风”突出问题，全面从严治党不断深入。坚定不移贯彻落实中央决策部署和省市委工作要求，认真执行市人大及其常委会决议、决定，自觉接受人大法律监督、政协民主监督、社会舆论监督，虚心听取各民主党派、工商联、无党派人士和社会各界意见，积极办理人大代表建议和政协委员提案。完成全市政务服务热线整合及“12345”新平台建设，开展“我向市长说句话”网民建言征集，努力解决群众关注的矛盾问题，法治政府建设和为民服务水平进一步提升。

各位代表！过去的一年，在宏观环境复杂严峻的形势下，全市经济社会发展克服各种困难，实现了新进展、取得了新成绩。这得益于省委、省政府和市委的坚强领导，得益于市人大、市政协和社会各界的有力监督和大力支持，得益于全市广大干部群众团结一致、迎难而上、负重奋进。在此，我代表市人民政府，向全市广大干部群众，向各民主党派、工商联、无党派人士、各人民团体及社会各界人士，向中央、省驻淄单位、驻淄部队和武警官兵、政法干警，向海内外所有关心支持淄博改革发展的同志们、朋友们，表示衷心的感谢和崇高的敬意！

同时，我们也清醒认识到，在发展道路上还面临不少困难和制约，主要是：外部环境依然复杂严峻，经济运行面临诸多不稳定不确定因素，稳增长压力持续较大；区域经济内生动力还不强，制约高质量发展的体制机制障碍仍然较多，“双招双引”质效不够高、重大新兴产业项目偏少、民间投资不够活跃、产业层次偏低、自主创新能力不强等瓶颈尚未根本突破，新旧动能转换“空笼期”问题比较突出；结构性污染治理任务艰巨，生态环境改善的基础较为脆弱，弥补历史欠账和解决遗留问题需要付出艰苦努力；区域之间、城乡之间发展依然不平衡不协调，群众关心的教育、医疗、就业、养老、扶贫、社会保障等领域还有不少短板；安全生产和风险防范压力时时存在，影响社会和谐稳定的潜在因素日益增多，社会治理面临许多新课题新挑战；政府自身建设还不能很好地适应新形势新要求，有的部门单位和机关干部思想观念滞后、专业能力不足、担当意识不强，缺乏干事创业的精气神，与建设人民满意的服务型政府还有较大差距。对于这些问题，我们一定高度重视，认真加以解决。

二、2019年经济社会发展目标任务和工作重点

2019年是中华人民共和国成立70周年，是决胜全面建成小康社会第一个百年奋斗目标的关键之年。我们要坚持以习近平新时代中国特色社会主义思想为指导，深入学习贯彻党的十九大和十九届二中、三中全会以及习近平总书记视察山东重要讲话、重要指示批示精神，坚持稳中求进工作总基调，以供给侧结构性改革为主线，紧紧围绕落实新发展理念、推动高质量发展，按照市委“一个目标定位、四个着力建设、十个率先突破”总体思路和工作布局，进一步稳就业、稳金融、稳外贸、稳外资、稳投资、稳预期，着力在推动新旧动能转换重大项目建设、“双招双引”和建设对外开放新高地、新型工业化强市建设和现代服务业发展、文化名城建设、生态淄博建设、现代化组群式大城市建设、乡村振兴和脱贫攻坚、民生社会建设、全面深化改革等方面率先突破，加快推进淄博转型发展、全面振兴、走在前列，为全面建成小康社会收官打下决定性基础，以优异成绩庆祝新中国成立70周年！

全市经济社会发展预期目标建议为：地区生产总值增长6.5%左右，一般公共预算收入增长5%左右，固定资产投资增长6%左右，社会消费品零售总额增长8.5%左右，城镇居民和农村居民人均可支配收入分别增长7%左右和7%以上，全面完成省下达的节能减排约束性指标。重点做好七个方面的工作：

（一）以只争朝夕的干劲，奋力加快新旧动能转换。面对艰巨繁重的转型发展任务和不断加剧的区域竞争态势，大力强化坐不住、等不起、慢不得的紧迫感责任感，牢牢把握以“四新”促“四化”，聚焦做好“高新轻绿”四篇文章，聚力培植“四强”产业，加快构建“753”现代产业体系。

一是加速工业高质量发展。启动工业新旧动能转换三年攻坚，聚焦新材料、智能装备制造、新医药、电子信息“四强”产业做加法，在壮大现有骨干企业的同时，布局实施高端医药制剂、智能医疗装备、精密数控机床、稀土新材料、智能机器人、新能源电池、激光设备、智能传感器等一批新兴产业项目，“四强”产业占全市工业比重年内力争提高到40%。围绕绿色化工、机械制造、新型建材、特色纺织、轻工、陶瓷琉璃等六大优势传统产业，集中抓好总投资960亿元的321个年度重点技改项目，对800家以上规上企业开展高端化、智能化、绿色化技术改造，推动3000家以上企业“上云”，在提高技术水平、扩大优质产能、更新换代产品、延伸产业链条等方面实现新突破。坚持做好“腾笼”减法，持续推进钢铁、焦化、煤电、基础化工等行业落后和过剩产能出清。充分发挥我市新材料、机电装备等方面的比较优势，积极融入海洋强省和军民融合战略，搞好产业协作配套，组织实施一批示范项目，借势加快高质量发展。全力支持央属、省属驻淄企业创新发展，充分发挥其人才、技术、规模、市场等优势，加大技术改造和新上项目力度，在安全、环保和新旧动能转换上发挥龙头作用。

二是加力推动现代服务业提质增效。抓住用好国家促进形成强大国内市场的机遇，聚力发展现代物流、文化旅游、现代金融三大现代服务业，突出抓好157个服务业重点项目和20家创新型服务业企业，年内服务业增加值增长7%左右。加快发展平台经济、创意经济、楼宇经济，持续推进11个重点物流园区建设，着力发展智慧物流。强化淄博新区商务金融中心集聚功能，提升齐鲁股交中心及齐商银行、农商行、招金期货、齐鲁农产品交易中心等法人机构运营水平，增强金融服务实体经济能力。发展壮大卓创资讯、齐鲁云商、华润物联网产业园、华云大数据小镇、海尔COSMO等智慧平台，积极培育工业设计、软件信息、工业互联网等制造业服务提供商，促进先进制造业与现代服务业深度融合。发展壮大健康养老、教育文化、旅游休闲、信息通讯等生活性服务业，推动批发零售、住宿餐饮等传统业态转型升级，努力增加优质服务供给、满足消费升级需求。

三是深入实施创新驱动战略。以创建国家创新型城市为统领，着力推进“两城一谷”建设，组织实施山东理工大学科技园等80个校城融合发展项目，加快建设科学城科创中心、MEMS产业创新园、齐鲁新材料中试基地等研发创新平台，切实深化与中国航天科工集团、中国电子科技集团、北京石墨烯技术研究院等大院大所合作，加快洽谈引进高等院校，年内新增省级以上研发平台、院士工作站等30家以上。突出企业和行业联盟创新主体作用，建设市知识产权运营公共服务平台，高新技术企业数量增长15%以上，研发投入占比达到2.51%以上。着力完善“双创”平台融资、研发、咨询服务等综合功能，构建要素完备的创业创新生态链。

四是强力推动产业集约集聚。深入开展“园区改革建设发展突破年”，完善省级经济开发区和重点园区基础设施，加快建设仓储物流、融资租赁等服务平台，年内建设标准厂房100万平方米以上。全面落实“管委会+公司+基金”运行模式，探索建立针对开发区的高效审批机制，推行“亩均税收”领跑者激励制度。统筹全市重点园区产业布局，实行招商项目“飞地政策”，推动“四强”及其细分产业加快实现园区化布局、集群化发展。强化重点化工园区承载功能，分类推进化工企业转型升级。集中抓好建陶、耐火材料、微电机、家具厨具、智慧厨电等特色产业集聚区，提升传统产业智慧化集约化水平。

五是奋力在“双招双引”上发力突破。扎实开展“四大招引工程”，突出以企招商、委托招商、基金招商，高水平举办境内外专题招商活动，全力引进世界500强和行业领军企业。建立全市招商信息共享平台，完善招商项目“绿色通道”和全程服务机制，年内引进过亿元产业项目170个以上，“四强”产业项目占比不低于60%。全面落实“人才新政23条”，鼓励市内高校毕业生留淄就业创业，实行高层次人才“一站式”服务，新增院士、“千人计划”专家等高端人才30名以上。加强落户安居、教育医疗、金融支持等服务保障，充分释放人才的“含金量”。

六是培育壮大转型发展优质主体。组织实施年度投资1000亿元的323个市重大项目，积极推行审批代办制度和资源要素跨区域统筹，无需新增用地项目上半年全部开工，新增用地项目10月底前全部开工。支持“双50强”和行业骨干企业跨越发展，鼓励企业通过上市挂牌、引进战略合作者、实施海内外并购做精做强主业。全面落实各级支持中小微企业发展政策措施，完善培训、咨询、代理等公共服务体系，省级“专精特新”企业达到270家。继续实施“十万企业家三年培训计划”和“高技能人才十年培训计划”，全年精准培训2万人和5000人以上。加大品牌培育力度，推进管理对标行动，引导更多企业走上创新驱动、品牌引领、质量振兴的内涵发展道路。

（二）以抓铁有痕的力度，坚决打好三大攻坚战。进一步强化底线思维、问题导向，推动三大攻坚战取得重大进展，为淄博转型发展、全面振兴、走在前列打开新局面。

一是把全力以赴打好污染防治攻坚战作为重中之重。坚持“绿水青山就是金山银山”，深入实施“四减四增”三年行动，以更高的标准、更严的要求、更实的举措，攻坚克难，精准施策，促进生态环境质量持续改善。加力突破大气污染治理瓶颈。扎实推进“1+4”专项行动，取缔35蒸吨/小时（不含）以下燃煤锅炉，关闭6台燃煤小机组，完成14万户农村清洁取暖改造任务，煤炭消费总量控制在2900万吨以内。精准实施石化、有机化工、包装印刷等行业污染点源治理，加快淘汰国Ⅲ重型柴油车。推动“公转铁”货物运输方式调整，加强骨干道路、建筑工地等领域扬尘污染治理，年内全市空气质量优良天数达到60%，“蓝天白云、繁星闪烁”天数达到80%。持续深化水污染防治。深入落实河（湖）长制，推进小清河淄博段治理工程，完成猪龙河篷河段综合整治，健全黑臭水体、黑臭坑塘排查治理长效机制。加严企业污水排放标准，推动工业聚集区加快建设污水集中处理设施，保障全流域水体稳定达标。扎实开展地下水污染专项治理，突出抓好化工园区、化工企业周边地下水检测整治，坚决消除风险隐患。实施光大水务一分厂搬迁及二分厂、三分厂提标改造，城镇污水处理厂稳定达到一级A标准。完成刘征水源地规范化建设，以最严的措施抓好水源地管理，确保城乡饮水安全。加大生态修复力度。加强重点生态功能区和省级自然保护区建设管理。推进重点河流和骨干道路生态廊道建设，完成71座已关闭露天矿山生态恢复治理和11处绿色矿山创建。开展固废危废填埋排查整治专项行动，建设临淄危险废物综合利用及处置中心，完善重点工业园区危险废物集中处置设施，年内基本消除超期贮存危险废物，各区县对固体废物实现集中处置。组织开展土壤环境质量详查，推进化肥农药减量使用，切实防治面源污染。健全污染防治长效机制。坚定不移抓好中央、省环保督察和生态环境部强化督查反馈问题整改，彻底解决一批重点环境问题。完成全市“三线一单”编制，加快推行生态补偿机制。加大联合执法、“刑责治污”力度，提升网格化、信息化、常态化监管水平，对环境违法行为坚决做到“零容忍”。

二是巩固提升脱贫攻坚成果。坚持精准扶贫精准脱贫，深化国家扶贫改革试验区建设，进一步完善重点（薄弱）村“五通十有”设施，集中抓好“一产业两收入”，确保贫困群众稳定实现“两不愁三保障”。加快黄河滩区17个村庄脱贫迁建，到2020年切实解决防洪安全和安居乐业问题。开展扶志扶智行动，增强贫困群众脱贫内生动力。推进扶贫开发政策和农村低保制度衔接与融合试点，完善干部挂包、结对帮扶、孝善扶贫、资产收益扶贫等制度，构建稳定脱贫长效机制。对没有列入省市重点贫困村的经济薄弱村进行全面排查，凡是没有纳入省市重点贫困村的薄弱村全部纳入2019年扶贫工作重点，确保所有贫困村和贫困户都得到帮扶、达到脱贫目标。

三是持续打好防范化解重大风险攻坚战。大力深化重点担保圈综合治理，有效实施预警防范和分类处置，努力实现不良贷款余额和不良贷款率“双下降”。严厉打击逃废银行债务、非法集资、金融诈骗等违法行为，持续优化区域金融生态。坚持严控增量、消化存量，既用足用好国家较大幅度增加地方政府专项债券规模的机遇，又坚决杜绝违规举债，做到依法举债、适度举债、合理举债。

（三）以传承创新的实践，不断激发文化发展活力。突出优秀传统文化创造性转化、创新性发展，深度挖掘、科学利用优势资源，打造多元转化载体，增加优质产品供给，努力实现厚植底蕴、提升品位、彰显特色有机统一，推动文化名城建设迈出新步伐。

一是切实增强齐文化影响力。以建设齐文化传承创新示范区为统领，加快推进齐国故城考古遗址公园、齐古城、稷下学宫等核心区重点项目。提升世界足球文化高峰论坛、“起源地杯”国际青少年足球锦标赛举办水平，积极争取在国内外重大足球赛事中更多植入蹴鞠元素。高水平办好齐文化节，以更大力度创新推动齐文化资源产业化和传播现代化，切实打响“齐国故都”城市品牌。

二是扎实推进文化与产业深度融合。加快全域旅游城市创建，依托丰富多彩的地域特色文化，深入实施文旅融合八大工程，集中抓好华侨城旅游综合体、陶琉古镇、中华传统民俗文化创意园等文旅融合重点项目，支持周村古商城创建5A景区，优化提升10处乡村旅游连片发展区。打造五阳湖体育休闲小镇、国井白酒文旅小镇、马踏湖文旅小镇等特色小镇，培育工业旅游、健康旅游新业态。建设市旅游公共服务中心和全市智慧旅游云平台，完善“快进慢游”路网体系，加强景区公共服务配套。深化与国内外大型文旅集团合作，加快发展创意设计、影视制作、数字出版等文化业态，不断增强文化产业规模实力。

三是持续提高城市文明水平。深入推进新时代文明实践中心建设，广泛开展践行社会主义核心价值观和文明创建活动，巩固提升文明城市建设成果。挖掘提升焦裕禄纪念馆、原山艰苦创业教育基地、马鞍山等红色文化资源，加快建设焦裕禄干部教育学院，打造全国知名的党员干部教育基地。抓好市文化中心管理运营，塑造高品位、地标性城市客厅。推进淄川、博山、临淄等区县文化中心建设，实现公共文化数字化建设全覆盖。组织开展庆祝建国70周年主题文化活动，扎实办好文化惠民实事，让人民群众精神文化生活更加丰富、更加充实。

（四）以统筹融合的路径，着力提升城市现代功能。以建设现代化组群式大城市为目标，按照“四位一体、组群统筹、全域融合”城市工作思路，扎实推进规划科学化、建设精品化、管理精细化、运营高效化，不断增强城市承载力和竞争力。

一是进一步优化城市空间布局。加快城市总体规划、新一轮土地利用总体规划编制工作，统筹编制市域空间规划。将淄川区纳入“四位一体”布局，推动张店（含高新区）、周村（含淄博经开区、文昌湖区）、淄川、桓台一体化发展。突出淄博新区、淄博经开区、文昌湖区、科学城、主城区南部区域等重点功能区块，实施一批基础设施、公共服务、新兴产业和生态治理项目，加快各区域旧村搬迁改造，着力打造“四位一体”关键支撑。继续推进张店东北部、东南部区县关联区域，以及桓台马桥和高青高城等边界关联片区互联互通、融合发展。实施峨池路、岭博路、博幸路建设改造以及供热、供气、物流等一批一体化基础设施项目，切实加快淄川、博山统筹发展。开工建设沾沂高速淄博段、上海路北延、跨小清河大桥等联通工程，促进沂源、高青与其他区县融合发展。

二是着力增强承载带动功能。实施北京路南延、张南路南延、共青团路和金晶大道提升改造等城市骨干路网工程，加快火车站南广场改造建设。集中推进G309青兰线、S229沂邳线改建等交通重点工程，完成济青高速、滨莱高速淄博段改扩建及4座城市立交项目。开工张博铁路电气化改造，完成城市轨道交通国家立项，积极做好滨淄临高铁、济淄潍高速、济南—高青高速、小清河复航等项目前期工作。组织实施电网结构优化、中心城区供水系统改造等公用设施项目。着力培育新生小城市、重点示范镇和特色小镇，力争户籍人口城镇化率提高1个百分点。

三是切实提升精细化管理水平。突出全覆盖、全时段精细管理，建立“大城管”管理体制，用力量下沉解决缺少监管的问题；提高环卫保洁市场化水平，用市场机制解决无人干活的问题；加大数字化、智慧化管理力度，用信息化手段解决无法问责的问题，切实把背街小巷、老旧小区、集贸市场、城乡结合部管住管好，彻底解决脏乱差问题。深入开展济青高铁沿线等路域综合整治，实施高速公路出入口绿地提升工程，开展违法建设、“大棚房”治理“清零”行动。对重点区域及标志性建筑进行亮化提升，打造富有韵味的城市夜景。完成“城市双修”全国试点任务，扎实推进“三改三建”工程，改造棚户区4573套、老旧小区项目40个、背街小巷14条，新改建便民农贸市场10处、城市公厕35座、城市公共停车场19处，进一步营造功能完善、优美舒适的人居环境。

（五）以精准务实的举措，深入实施乡村振兴战略。全面落实全市乡村振兴规划和五个专项工作方案，健全城乡融合发展体制机制，统筹推进乡村产业振兴、人才振兴、文化振兴、生态振兴和组织振兴，努力打造生产美产业强、生态美环境优、生活美家园好的乡村振兴齐鲁样板中的淄博特色板块。

一是推进现代农业产业发展。围绕发展农业“新六产”，拓展乡村旅游、农村电商“两大亮点”，逐一明晰全市自然村产业重点和发展方向，积极支持涉农电商平台、“新零售”等新业态加快发展，打造一批“一村一品”示范村镇，着力构建村有主业、镇有特色、县（区）成规模的农村产业格局。开展粮食绿色高质高效创建，切实保障粮食安全。培育壮大沂源苹果、高青黑牛等特色农产品优势区，“三品一标”农产品达到320个。实施“百企建百园”工程，大力引导工商资本进入乡村，建设临淄现代农业产业园、得益乳业生态种养基地、沂河源田园综合体等特色园区，推进农业与文化旅游、健康休闲等产业深度融合。

二是持续推进美丽乡村和文明乡村建设。统筹实施美丽乡村示范创建和“百村示范千村整治”工程，开展农村人居环境整治三年行动，年内打造乡村振兴示范村100个左右、重点村1000个左右。实施水安全保障规划，提升农村饮水安全工程，加强水利等领域灾后重点防灾减灾设施建设。巩固城乡环卫一体化成果，抓好博山区省级农村生活垃圾分类试点。完善生活污水处理、清洁供暖等基础设施，实现农村旱厕应改尽改、长效管护。推进“四好农村路”建设，完成330公里农村公路、21座危桥、1044个村庄道路硬化“户户通”改造提升任务，新增农村客运站点1314个。积极打造城乡教育联合体，发展“互联网+教育”，让优质教育资源惠及更多农村孩子。全面完成基层医疗卫生服务机构标准化建设，“第一村医”覆盖到1500个村庄。实施乡村文化振兴“六大工程”，建成村级（社区）综合文化服务中心示范点300个左右、“乡村记忆”博物馆60个左右。加强乡村法治建设，深化乡村文明行动，提高乡村德治水平。

三是激发农业农村发展活力。深化农村集体产权制度改革，加快农村闲散土地盘活利用，发展壮大农村集体经济。稳妥推进承包地和农村宅基地“三权分置”改革，完善农村土地流转交易和抵押融资两个平台，家庭农场、农民合作社等新型经营主体突破9000家。整合建立乡村振兴资金池，健全农村金融和涉农保险体系，推动编制资源向镇（街道）倾斜，年内培育新型职业农民4000人以上，为乡村振兴提供坚实支撑。

（六）以攻坚克难的锐气，持续深化改革扩大开放。40年改革开放的伟大实践启示我们，必须始终坚定改革开放的信心决心，着力突破制约高质量发展的障碍藩篱，着力提升开放型经济的质量水平，不断激发老工业城市转型发展内生动力。

一是加力深化重点领域改革。认真贯彻省、市委决策部署，按时完成市县机构改革任务。深入推进投融资体制改革，设立市新旧动能转换基金，深化与金融机构、投资机构和境内外社会资本合作，年内实现“四强”产业子基金全覆盖。充分借助多层次资本市场，推动企业上市挂牌，力争2家企业实现上市。深化资源要素市场化配置改革，探索建立节能量、碳排放权、排污权、水权交易机制；全面开展“亩均论英雄”专项行动，对全市规上工业企业逐一核算亩产效益；加强“批而未供”“供而未用”土地清理整顿，盘活存量资源、推动集约发展；集成市级相关政策，集中投放到新旧动能转换重点领域和关键环节。深化国资国企改革，加快实现从管企业向管资本转变，促进国有资本保值增值。深化财政体制改革，完善市对区县财政体制，加大对基层财政的转移支付力度；加强预算绩效管理，构建“全方位、全过程、全覆盖”的预算绩效管理体系。积极落实国家税制改革部署，实质性、普惠性降低企业负担。做好全国第四次经济普查，加快形成助推高质量发展的统计体系。推进“互联网+各领域工作”改革，更加有力地提高效率、降低成本，打通堵点、破除瓶颈。

二是大力发展开放型经济。积极应对中美贸易摩擦，加大新兴市场开拓力度，加快发展外贸综合服务、跨境电商等新业态，扩大机电及高新技术产品出口，年内进出口10亿元以上企业达到13家，品牌出口占比超过30%。深度参与“一带一路”建设，重点抓好3个境外园区和10个在建项目。加快淄博内陆港建设，扩大“齐鲁号”欧亚班列辐射范围，推进淄博保税物流中心申建综保区，打造贸易物流双向大通道。

三是着力优化营商环境。坚持以市场主体和群众满意为导向，按照“一不三一”标准，聚焦“一次办好”改革，持续深化12个优化营商环境专项行动，深入推进网上“不见面审批”，年内基本实现“一门式、一窗式、一网式”办理。严格落实各级支持实体经济、民营经济发展政策措施，加强政策宣传和效果评价，切实增强微观主体活力。深化政银企合作，鼓励银行等金融机构创新产品和服务，完善政府性融资担保体系，有效破解中小企业融资难、融资贵、融资慢问题。持续开展增加企业负担专项治理，严格规范涉企检查、收费以及第三方评估评审，最大限度降低营商成本。充分激发和保护企业家精神，构建亲清新型政商关系。深入推进“信用淄博”建设，实现守信联合激励、失信联合惩戒，打造公平竞争的市场环境。

（七）以为民服务的自觉，大力加强民生社会建设。坚持以人民为中心的发展思想，完善制度，守住底线，切实解决群众最关心最直接最现实的利益问题，不断增强人民群众的获得感幸福感安全感。

一是进一步提高就业和社会保障水平。把稳就业摆在突出位置，实施就业优先政策，加强创业载体建设和创业贷款支持，促进高校毕业生、农民工、退役军人、去产能企业分流职工等重点群体实现更高质量就业，全年城镇登记失业率控制在4%以内。扎实推进“全民参保计划”，稳步提高各类保障补助标准，积极扩大住房公积金制度覆盖面。统筹城乡社会救助体系，全面落实特困人员基本生活和照料护理两项标准，更加关爱农村留守儿童、残疾人等困难群体。完善养老护理体系，加强敬老院规范化管理和安全隐患治理，日间照料中心覆盖90%以上城市社区，农村幸福院等互助养老服务设施覆盖50%以上村庄。

二是协调发展教育卫生等社会事业。积极创建国家义务教育优质均衡县（区），抓好淄博新区高中、淄博十七中新校等项目建设，实施43所中小学新改扩建，其中中心城区新开工9所，建立完善解决“大班额”长效机制；推进第三期学前教育行动，新改建普惠性幼儿园80处以上；增强高等院校自主发展活力，支持淄博职业学院创建国家特色高水平高职院校。深入推进健康淄博建设，持续深化医药卫生体制改革，加快建设市公共卫生中心，全面投用市中心医院西院区等重点项目；落实“健康中国人”行动计划，完善四级“互联网+医疗健康”体系，2020年基本完成区县域医共体建设；加强疫苗流通和预防接种管理，开展医疗领域突出问题专项治理，严厉整治非法行医、保健品会销、虚假医药广告、骗取医保资金等不法行为。实施餐饮质量安全提升和“品质鲁药”工程，争创国家食品安全示范城市。促进体育产业、群众体育与竞技体育协调发展。开展新一轮双拥模范城创建，支持国防和军队建设，落实各项优抚政策，切实做好军转安置和退役士兵服务保障工作。

三是不断加强和创新社会治理。深化法治淄博、平安淄博建设，深入推进扫黑除恶专项斗争，统筹抓好“雪亮工程”、网格化管理、矛盾纠纷多元化解、综治中心建设、社区治安综合治理精细精准工程“五位一体”建设，年内建成100个以上平安示范小区，70%以上城市社区服务中心达到规范化标准，城市社区专职网格员覆盖率达到100%。压紧压实安全生产责任，深入开展重点行业领域风险隐患排查治理，健全应急管理处置机制，确保社会大局和谐稳定。

三、努力建设人民满意的服务型政府

新时代、新使命、新征程，对政府工作提出了新的更高要求。全市各级政府必须牢记宗旨使命、自觉恪尽职守、奋力开拓创新，以忠诚干净、认真专业、担当作为的实际行动，努力建设人民满意的服务型政府。

坚持从严治政。把旗帜鲜明讲政治贯穿于政府工作全过程，深入学习贯彻习近平新时代中国特色社会主义思想和党的十九大精神，树牢“四个意识”，坚定“四个自信”，坚决做到“两个维护”，巩固增强政治建设的思想自觉和行动自觉。严守政治纪律和政治规矩，不折不扣贯彻执行党中央决策部署和省市委工作要求，确保政令畅通、令行禁止。认真开展“不忘初心、牢记使命”主题教育，切实加强学习型政府建设，全面提升公务员队伍政治素质、专业能力和实干精神。

坚持依法行政。落实重大行政决策法定程序，对关系转型发展和群众利益的重要决策事项，更加主动地听取市场主体、社会公众及专业人士意见，提高决策科学性和精准度。以机构改革为契机，不断强化市场监管、社会治理、公共服务、生态保护等职能，推动资源、服务、管理向基层下沉。自觉接受人大及其常委会法律监督、人民政协民主监督、社会和舆论监督，高质量办理人大代表建议和政协委员提案。完善“12345”政务服务平台功能，继续办好“我向市长说句话”网民建言征集，使政府工作更加充分体现人民意愿。

坚持担当勤政。认真落实市委“三个转变、一个强化”要求，带着责任和感情深入基层一线，面对面了解实情，实打实破解难题。把抓落实作为政府工作的生命线，深入开展“工作落实年”，推进工作项目化、项目清单化、清单责任化，作出的承诺坚决兑现，定下的事情一抓到底。坚持作风建设永远在路上，驰而不息纠治形式主义、官僚主义等“四风”突出问题，切实整治“四大重症”和不作为、慢作为等作风顽疾。健全激励约束机制，为担当者担当、为负责者负责，凝聚形成想干事、能干事、干成事的强大合力。

坚持廉洁从政。严格落实全面从严治党从严治政重大责任，抓严抓实政府系统党风廉政建设，坚决整治群众身边腐败和作风问题。突出公共资金、国有资产资本、工程建设、政府采购等重点领域，深入排查廉政风险隐患，完善公共资源交易等长效机制。牢固树立过紧日子的思想，严格控制“三公”经费和一般性支出，把有限资源和财力更加充分地用在推动发展、改善民生上。强化审计监督，扩大政府信息公开范围，让权力在阳光下运行，使广大公务人员自觉做到知敬畏、存戒惧、守底线，切实营造风清气正的政治生态。

各位代表！新时代绘就新蓝图，新使命呼唤新担当。让我们高举习近平新时代中国特色社会主义思想伟大旗帜，更加紧密地团结在以习近平同志为核心的党中央周围，在中共淄博市委的坚强领导下，不忘初心、牢记使命，埋头实干、锐意进取，为决胜全面建成小康社会，谱写老工业城市转型发展、全面振兴、走在前列新篇章而努力奋斗！
